# Hot Water Treatment for Post-Harvest Disinfestation of *Bactrocera dorsalis* (Diptera: Tephritidae) and Its Effect on cv. Tommy Atkins Mango

**DOI:** 10.3390/insects12121070

**Published:** 2021-11-29

**Authors:** Nelson L. Mwando, Shepard Ndlela, Rainer Meyhöfer, Sevgan Subramanian, Samira A. Mohamed

**Affiliations:** 1 International Centre of Insect Physiology and Ecology (icipe), Nairobi P.O. Box 30772-00100, Kenya; sndlela@icipe.org (S.N.); ssubramania@icipe.org (S.S.); sfaris@icipe.org (S.A.M.); 2 Institute of Horticultural Production Systems, Section Phytomedicine, Applied Entomology, Leibniz Universität Hannover, Herrenhäuser Straße 2, 30419 Hannover, Germany; meyhoefer@ipp.uni-hannover.de

**Keywords:** quarantine treatment, *Mangifera indica*, nonchemical, phytosanitary, oriental fruit fly, physicochemical parameters

## Abstract

**Simple Summary:**

The oriental fruit fly *Bactrocera dorsalis* is a major quarantine pest in sub-Saharan Africa that threatens mango production and international trade. In this study, we developed a hot water treatment (HWT) protocol for the post-harvest disinfestation of *B. dorsalis* and assessed its impact on cv. Tommy Atkins mango quality parameters after treatment. First, we established the rate of development of the immature stages of *B. dorsalis* in cv. Tommy Atkins mango and then determined their heat tolerance. The third-instar larva was found to be the most heat tolerant of the immature stages. The study demonstrates that a hot water treatment schedule of 46.1 °C for 72.63 min can lead to complete mortality of the most heat-tolerant stage of *B. dorsalis* in cv. Tommy Atkins mango. Furthermore, we carried out large-scale confirmatory trials to validate our hot water treatment schedule, and none of the 59,120 most heat-tolerant larvae treated survived. Our protocol guarantees effective quarantine security with no adverse effect on the quality of cv. Tommy Atkins mango fruit and can be commercially adopted to promote and increase mango exports to lucrative markets abroad.

**Abstract:**

Mango production and trade in sub-Saharan Africa is hampered by direct damage and the high quarantine status of *B. dorsalis* and the paucity of effective post-harvest phytosanitary treatments. The current study reports the development of a quarantine treatment protocol using hot water to disinfest *B. dorsalis* and assess its effect on cv. Tommy Atkins mango quality. We first determined the development of the eggs and all larval stages of *B. dorsalis* in cv. Tommy Atkins mango and used the information to establish a time–mortality relationship of the immature stages after subjecting infested fruits to a regimen of eight, time instances of hot water at 46.1 °C. Using probit analysis, we estimated the minimum time required to achieve 99.9968% mortality of each stage. Our results indicate that the egg was the least heat tolerant, followed by the first, second, and third instar. The time required to achieve 99.9968% control of the third instar in cv. Tommy Atkins mango (400–600 g) was determined to be 72.63 min (95% Cl: 70.32–74.95). In the confirmatory trials, the hot water treatment schedule of 46.1 °C/72.63 min was validated, and none of the 59,120 most heat-tolerant individuals treated survived. Further, there were no significant differences between hot water-treated and untreated mangoes recorded in weight loss, fruit firmness, pH, total soluble solids, moisture content, and titratable acidity eleven days post-treatment. These findings demonstrate an effectively optimum post-harvest disinfestation treatment against *B. dorsalis* in cv. Tommy Atkins mango that should be adopted commercially to facilitate access to profitable but strict export markets globally.

## 1. Introduction

In sub-Saharan Africa (SSA), horticultural ventures play a key role in many countries’ economies, with mango production being one of the key enterprises that contribute immensely to the sector [1,2]. However, the production and trade of mango in the region do not meet the global market demands and standards chiefly due to infestation by quarantine fruit flies [3,4,5].

The infestation of mango with the oriental fruit fly *Bactrocera dorsalis* (Hendel) (Diptera: Tephritidae) has been reported to cause approximately 30–80% damage through feeding on the pulp of the fruit and, in some cases, up to 100% loss if the fly is left unmanaged [6,7,8,9]. In addition to the direct damage, interceptions and quarantine restrictions hinder the export of the produce, limiting access to profitable markets [10,11]. Similarly, in countries with established populations of *B. dorsalis*, huge financial resources are spent yearly on the management of the pest, leading to increased cost of production [9,12,13]. The *B. dorsalis* is categorized as a quarantine pest of high importance by many regional plant protection organizations [14,15]. The organizations’ member countries have set strict phytosanitary measures to ensure quarantine security when importing fresh horticultural produce from countries with populations of *B. dorsalis* [16]. Imperatively, the National Plant Protection Organizations (NPPOs) across SSA endeavor to maintain production areas free from *B. dorsalis* in their respective countries.

In the effort to suppress fruit flies at pre-harvest, mango farmers in SSA are usually encouraged to use safe, environmentally friendly, and sustainable integrated pest management (IPM) options. These include spot application of food baits, the male annihilation technique, *Metarhizium anisopliae*-based biopesticide application, the releases of parasitoids (*Fopius arisanus* (Sonan) and *Diachasmimorpha longicaudata* (Ashmead)), and the use of orchard sanitation [9]. Studies have indicated that if at least two of the components are correctly and consistently applied, the IPM packages will be effective and significantly reduce mango damage caused by fruit flies in SSA [17,18]. However, the application of these management options, especially among smallholder farmers, is limited due to several factors, particularly resource constraints and lack of knowledge [10,19]. Therefore, pre-harvest insect pest management strategies on their own do not guarantee 100% efficacy, and often, the pests obstinately find their way in the fruits post-harvest. The detection of even a single quarantine pest, such as *B. dorsalis* larva, in incoming fresh produce may result in the instant destruction of the whole consignment at the expense of the exporter or may even provoke the ban of future shipments into destined markets [20,21,22,23,24]. Thus, to overcome trade restrictions, post-harvest phytosanitary treatment of mango fruits possibly infested with *B. dorsalis* is necessary to complement pre-harvest management tools.

The current post-harvest quarantine treatment methods commonly applied to control *B. dorsalis* include fumigation, radiation, insecticidal dipping, cold treatment, electromagnetic energy treatment, and hot vapor or hot water treatment [25,26]. Some of these methods have been shown to be effective depending on the host crop, albeit with challenges, including several safety- and health-related obstacles [27,28,29]. Among these methods, hot water treatment (HWT) stands out as an effective, viable chemical-free, and environmentally friendly phytosanitary option for *B. dorsalis* on mango [30,31,32]. 

The HWT technique involves immersing fruits in water at a specific temperature for a pre-determined time duration [33]. The temperature and time duration to be used in the heat treatment procedure are carefully determined by first establishing the thermal tolerance of the different stages of the target pest on the commodity and, second, by determining the minimum time required to kill at least 99.9968% of the most heat-tolerant stage of the pest [34,35]. There are several HWT quarantine treatment parameters developed against *B. dorsalis* in export fruits from sub-Saharan Africa. For instance, fruit packing sheds in Mozambique are currently implementing a HWT at 47 °C for 12 min, established for mature green mangoes destined for the South African export market [25]. In West Africa, a HWT that leads to a core temperature of 46.5 °C for disinfesting *Kent* mango cultivar against *B. dorsalis* was developed and recommended [36]. Additionally, a recent study by Ndlela et al. [37] revealed that a HWT at 46.1 °C for 68 min resulted in 100% mortality of the most heat tolerant third-instar larvae of *B. dorsalis* in *Apple* mango variety. The effective temperature and time of immersion, therefore, vary depending on several factors, including the fruit shape, weight, and cultivar [38]. On mango, most HWT procedures are effective at 46.1 °C, over specified periods for specific pests and cultivars [37,39]. Equally, consumer acceptability of fresh horticultural produce, including mango, is largely influenced by the fruit quality post-harvest. Thus, the present study aimed at developing a post-harvest treatment protocol for the disinfestation of *B. dorsalis* in cv. Tommy Atkins mango using hot water by (i) determining the development rate of *B. dorsalis* in the mango, (ii) testing the thermal tolerance of the immature life stages of *B. dorsalis*, (iii) estimating the minimum time required to achieve 99.9968% mortality of the most heat-tolerant stage, and, further, (iv) carrying out large-scale confirmatory tests. Lastly, we (v) assessed the physical and biochemical quality of cv. Tommy Atkins mango after treatment. 

## 2. Materials and Methods

### 2.1. Mass Rearing of Fruit Flies 

The mass rearing of the *B. dorsalis* flies used in this study followed the procedures described by Ekesi and Mohammed [40] at the animal rearing and quarantine unit of the International Centre of Insect Physiology and Ecology, Nairobi, Kenya. The field-collected adult flies were reared on an artificial diet (sugar and ultrapure grade enzymatic yeast hydrolysate) and water. The colony was continuously augmented with wild flies at three-month intervals during the two years of this experiment to avoid potential inbreeding depression or genetic divergence from wild populations.

### 2.2. Procurement of Experimental Mango Fruits cv. Tommy Atkins 

The cv. Tommy Atkins mango fruits used in this study were harvested from three different orchards, 3–5 ha in size, in Embu County (00°28′591″ S; 037°34′544″ E, 1327 m above sea level (asl)), Kitui County (01°05′516″ S; 038°01′177″ E, 1252 m asl), and Makueni County (01°47′402″ S; 37°21′473 E, 1226 m asl). Mango trees were treated against fungi as described in Ndlela et al. [37]. Before maturity, fruits were bagged in brown paper bags to prevent exposure to fruit flies as described in Ekesi et al. [3]. The maturity of the bagged fruits was assessed and determined using the conventional market indices, such as fruit life in terms of days after pollination, fruit shape, and size. The mature fruits were then harvested and sorted to ensure that only those weighing between 400 and 600 g, and free of disease, pests, and injuries, were used.

### 2.3. Hot Water Treatment Tank

**Figure 1:**
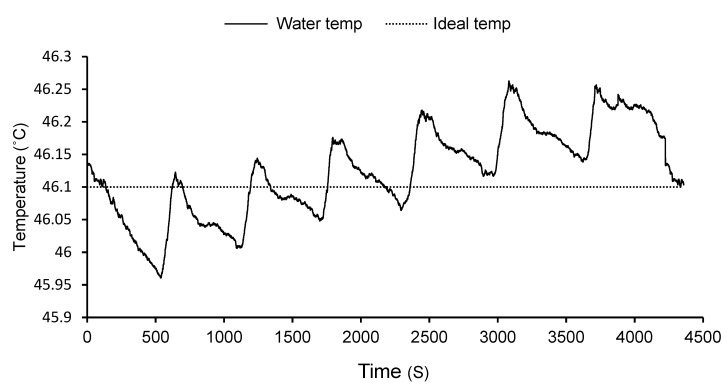
Mean temperature of water in hot water treatment tank recorded every second during the treatment process. The temperature range for this particular treatment was 45.96–46.26 °C with an average temperature of 46.12 °C.

An insulated stainless-steel tank (Desbro Engineering Ltd., Nairobi, Kenya) with a total volume of 1600 L, fitted with 16 heating elements with a total heating power of 48 kW and a pump (0.75 kw; 100 L/min) was used in the experiment. The two thermo-regulators fitted on the tank had digital temperature process controllers, with platinum resistance thermometer sensors with a stated accuracy of 0.5 °C. The hot water treatment equipment was also connected to a Grant Squirrel data logger (SQ2020-2F8) with 16 thermocouple probes (Tempcon instrumentation Ltd., Arundel, UK) to give temperature recordings throughout the treatment (Figure 1). Data from the logger could be downloaded to a laptop (software, Grant SquirrelView, version 5.1) as needed. 

### 2.4. Development Time and Heat Sensitivity of B. dorsalis in cv. Tommy Atkins Mango

Determination of the development rate and heat tolerance of the eggs and the first (1st)-, second (2nd)-, and third (3rd)-instar larvae of *B. dorsalis* in cv. Tommy Atkins mango was carried out according to Ndlela et al. [37], albeit with a few modifications. Briefly, a batch of 90 superficially cleaned fruits was infested and incubated at ambient conditions (25 ± 1 °C, 60–70% RH). Each day for nine consecutive days, 10 of the fruits were randomly picked from the batch and dissected, and 200 individuals were examined under a stereomicroscope to determine the development of the immature *B. dorsalis*. To determine the heat tolerance of the immature *B. dorsalis*, a group of twenty fruits harboring the respective immature stages of interest were picked at random from the holding crates and placed into one of eight perforated stainless-steel crates measuring 57 × 40 × 23 cm (Desbro Engineering Ltd., Nairobi, Kenya). The crates with infested fruits were immersed in a HWT tank for 8, 15, 23, 30, 38, 53, 60, and 68 min. The period between loading all the 8 crates was approximately 80 s. After 24 h, the treated fruits were dissected, and the numbers of live and dead target larvae were recorded. Fruits treated for eggs were kept for two more days and then dissected on the third day, and any hatched larvae were recorded. An equal number of infested mangoes were set aside as control to use as an estimation of the number of eggs and larvae in the treated batch. The experiment was replicated 7 times, and a total of 1120 fruits were treated for each immature stage. Time–mortality relationships were established, and from the data, the minimum time required to achieve 99.9968% mortality for each stage was estimated.

### 2.5. Large-Scale Confirmatory Tests

Fruits harboring third instars (the most heat-tolerant *B. dorsalis* in cv. Tommy Atkins mango with a net weight of up to 600 g) were subjected to large-scale HWT trials to validate the estimated minimum time (72.63 min) required to cause 99.9968% mortality of the treated larvae. The experiment was replicated 8 times, with a total of 1280 fruits (160 fruits per replicate) treated, and an equal number remained untreated (control). Thereafter, both the treated and untreated fruits were dissected, and the number of dead and live larvae was recorded.

### 2.6. Assessment of cv. Tommy Atkins Mango Quality after HWT

One hundred and sixty (160) mango fruits with uniform color, size, and firmness were randomly divided into two groups. The first group was subjected to a HWT for 72.63 min at 41.6 °C as described above. The other group was held untreated at ambient conditions (25 ± 1 °C). At least three mangoes were randomly picked from each batch and thereafter subjected to several tests to assess the impact of the treatment protocol on physical signs of heat injuries or accelerated skin color change, weight loss, pulp pH, fruit firmness total soluble solids, titratable acidity, and moisture content, from the first day and every second day for 11 days, following the methodology described in AOAC [41] and Padda et al. [42], with slight modifications. The experiment was repeated three times.

Briefly, physical changes due to HWT were assessed by looking for any signs of skin scorching, bruising, and other visible damage. A portable digital scale (Ohaus CS2000, Melrose, MA, USA) was used to determine weight loss by weighing the mangoes before and after treatment, and the results were expressed in percentages. To determine fruit firmness, a digital penetrometer (Turoni-53205, Forlì, Italy) equipped with an 8 mm diameter strut was used. The values were expressed in Newtons (N). The pH of the mango was measured by a digital pH meter (Orion 5 Star, Thermo scientific, Waltham, MA, USA) at ambient temperature using juice extracted directly from the pulp. Total soluble solids (TSS) were determined from pulp samples that were homogenized in a blender, thoroughly mixed, and filtered through a cheese cloth using a manual juicer, and then a drop was used to measure TSS content using a digital refractometer (Atago PR-101a, Cole-Parmer/Antylia Scientific, Vernon Hills, IL, USA). Titratable acidity (TA) was measured in the pulp through titration of an aliquot of 10 mL of the filtered extract against 0.1 N sodium hydroxide (NaOH) using phenolphthalein at 1% as an indicator. The data were expressed in percent of citric acid. Moisture content was determined by first recording the initial weight of the pureed sample in a pre-weighed aluminum dish. The samples were then dried in an oven (Nabertherm oven, RT-120 Lilienthal, Nabertherm, Lilienthal, Germany) overnight at 105 °C and weighed again. The moisture content was expressed in percentage. 

### 2.7. Data Analyses

The data on the development rate of immature stages of *B. dorsalis* were scored as a percentage to estimate the relative abundance of each stage over 9 days. Mortality data from the heat tolerance determination experiments were corrected for control mortality [43]. The data were then subjected to a generalized linear model of regression analysis with a probit function (dose.p function from MASS library) to determine the lethal time required to attain 50%, 90%, 99%, 99.9%, 99.99%, and 99.9968% mortality. To compare LT_99.9968_ values across the different stages, we first calculated the ratios of the LTs, and we then calculated the 95% confidence limits for these ratios as described by Robertson et al. [44]. Data from the validation tests were expressed as % mortality. Data on the selected physicochemical properties were first tested for normality by the Shapiro normality test and then analyzed by t-test. All analyses were performed using R software version 4.0.0 [45]. 

## 3. Results

### 3.1. The Development Rate of Bactrocera dorsalis in cv. Tommy Atkins Mango

During the incubation, the eggs started hatching 2 days after oviposition, and by day 3, more than 97% of the larvae were in the first instar. By days 5 and 6, 96.5% and 98% of the larvae, respectively, were in the second instar. By days 8 and 9, third-instar larvae accounted for between 99.5% and 100% of larvae in the fruit, with most mature larvae leaving the fruits to pupate on day 9. On this basis, the first, third, sixth, and eighth days were deemed to represent the eggs and the first, second, and third instars, respectively (Table 1). 

### 3.2. Heat Sensitivity of Bactrocera dorsalis in cv. Tommy Atkins Mango

The increase in the mortality of all the immature stages of *B. dorsalis* correlated with the increased treatment time (Table 2). The estimated LT_99.9968_ mortality for the egg stage and the first-, second-, and third-instar larvae was 45.90 min (range 44.47–47.33 min), 60.36 min (range 58.40–62.32 min), 62.93 min (range 60.93–64.94 min), and 72.63 min (range 70.32–74.95 min), respectively (Table 3). The third instar was, therefore, the most heat tolerant, while the egg was the most susceptible stage to the treatment schedule.

### 3.3. Large-Scale Confirmatory Trials

Based on the exploratory test results, large-scale confirmatory tests were performed on cv. Tommy Atkins mango fruits infested with the third instar for 72.63 min in 46.1 °C water, and of the 59,120 individuals treated, none survived. The mean natural mortality in the untreated control, consisting of 60,022 larvae, was 2.92% (range 1.62–4.29%) (Table 4). 

### 3.4. Changes in cv. Tommy Atkins Mango Fruit Quality Parameters after HWT

**Figure 2:**
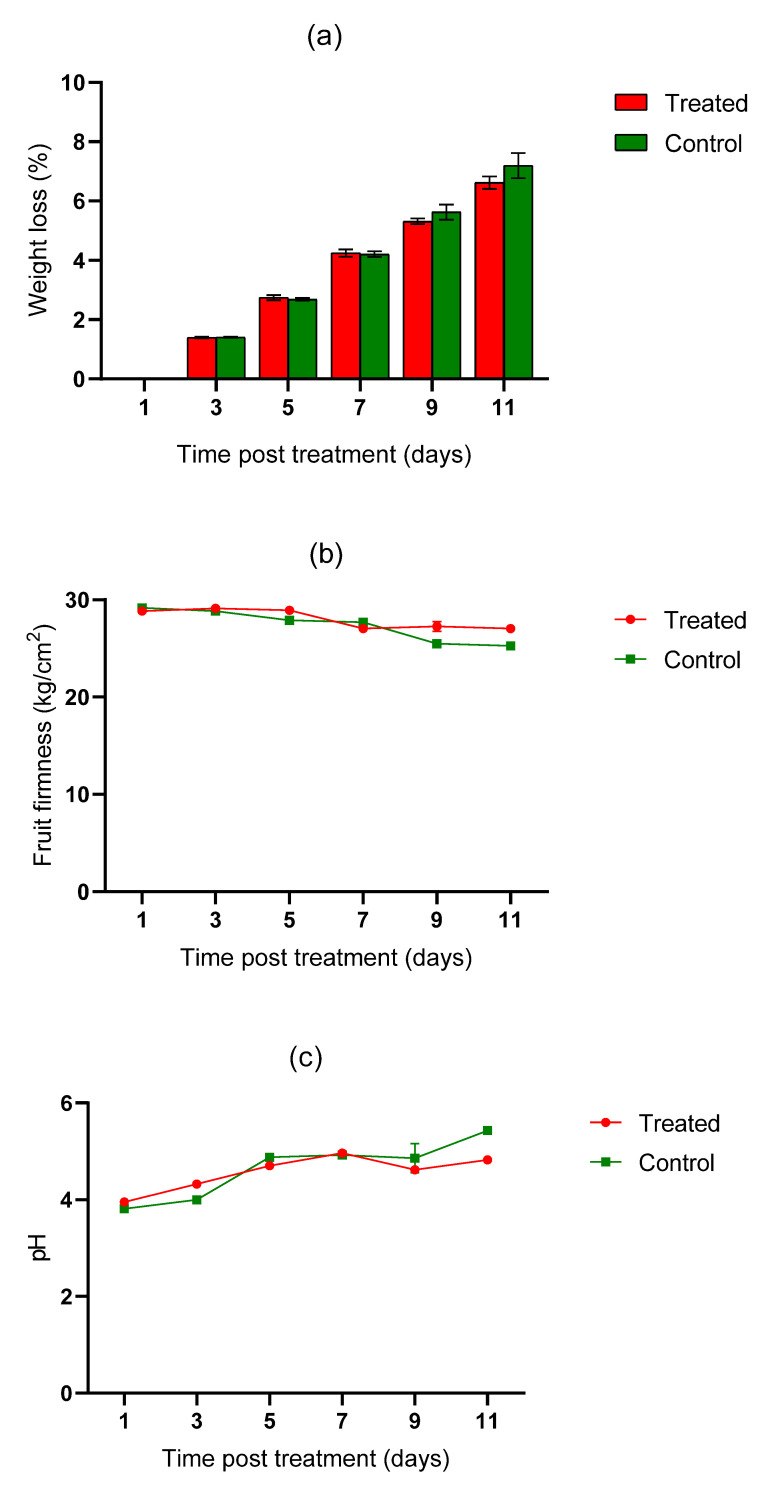

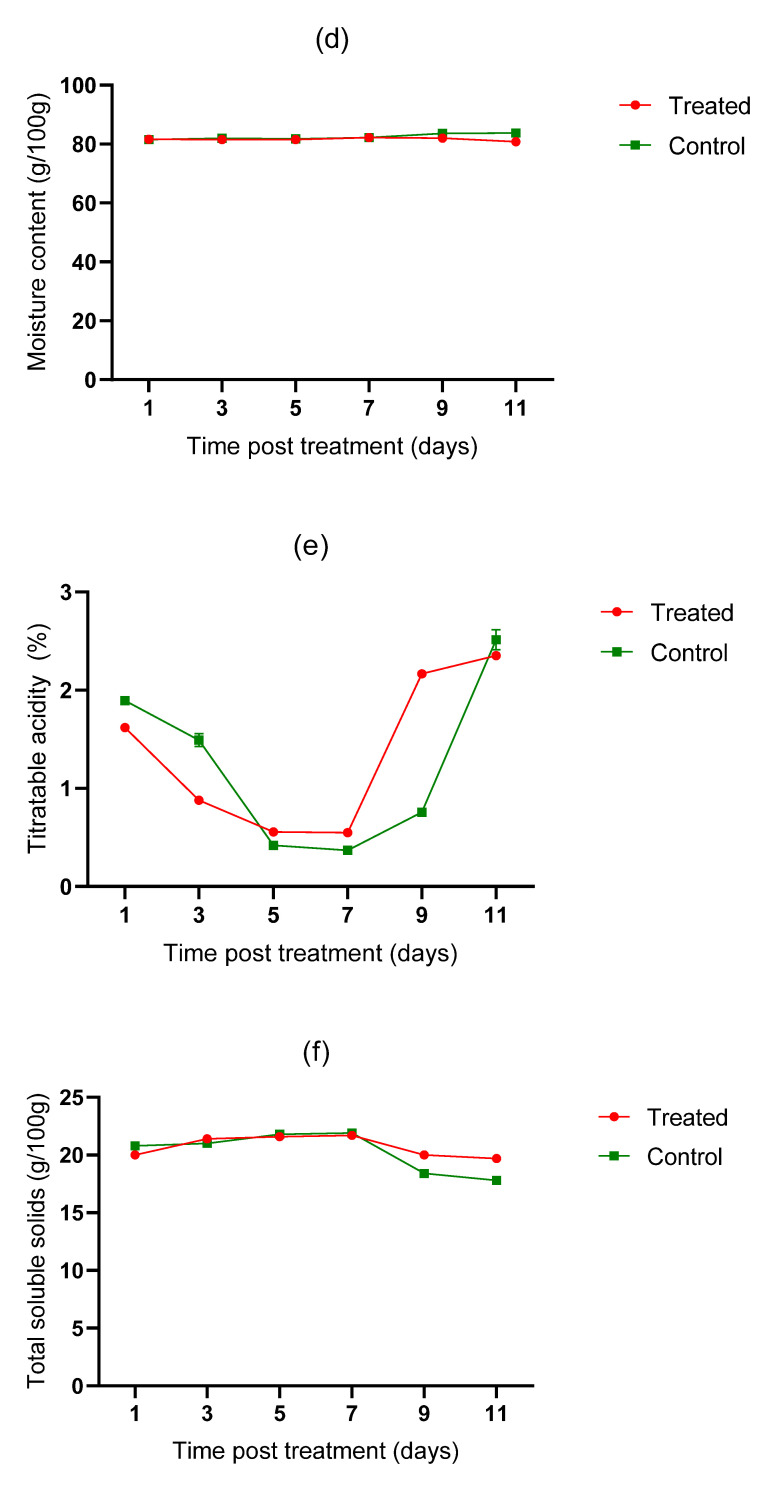
Changes in weight loss (**a**), fruit firmness (**b**), pulp pH (**c**), moisture content (**d**), titratable acidity (**e**), and total soluble solids (**f**) in cv. Tommy Atkins mango after HWT.

There were no visual signs of heat injuries or accelerated skin color development observed in either the untreated or heat-treated fruits over the 11 days post-treatment. Similarly, there was no significant difference between the treated and untreated mango fruits in terms of changes in weight loss (t = −0.089, df = 9.939, *p* = 0.931), fruit firmness (t = 0.775, df = 7.968 *p* = 0.461), pH (t = −0.293, df = 8.181, *p* = 0.777), total soluble solids (t = 0.556, df = 7.575, *p* = 0.595), titratable acidity (t = 0.556, df = 7.575, *p* = 0.595), and moisture content (t = 0.67, df = 9.898, *p* = 0.518) eleven days post-HWT (Figure 2a–f).

## 4. Discussion

Temperature and host quality are major factors that influence the developmental rate of insects, including tephritid fruit flies [46,47]. Similarly, the variety of the host plant has an impact on insect performance, including development [48]. In this study, the incubation period required for the *B. dorsalis* eggs in cv. ‘Tommy Atkins’ mango at ambient conditions (25 ± 1 °C, 65 ± 5% RH) to hatch was 2 days. This finding is comparable to results of previous studies that reported an average of 1.2 ± 0.02 days [49], 1–2 days [50], 2–3 days [37], and 3 days [51]. The slight variances in the above findings are probably due to differences in the incubation temperature, host material, and experimental procedure, which are known to influence the development and performance of all stages of *B*. *dorsalis* [47,52].

In different fruit fly species, thermal tolerance varies relative to developmental stage, fruit hosts, and cultivars [53,54,55]. In the current study, the time–mortality relationship (Table 2) revealed that the third instar was the most heat-tolerant stage of *B. dorsalis* in cv. ‘Tommy Atkins’ mango, followed by the second instar, the first instar, and the egg. The difference in heat response for different immature stages is a common phenomenon in tephritid fruit flies [47,56]. Our finding corroborates that of Ndlela et al. [37] and Hernández et al. [57], who carried out similar research on *B. dorsalis* in cv. ‘Apple’ mango and *Anastrepha sp.* in cv. ‘Ataulfo’ mango, respectively. The host and the location of the immature stage in the host fruit, therefore, influence their response to different treatments, including heat [58]. The less than a day-old eggs treated in our study were mostly small and, therefore, exceedingly susceptible to the treatment. Unlike the second- and third-instar larvae that reside in the very innermost part of the mesocarp, the first-instar larvae are small, mostly found immediately under the fruit exocarp, and are therefore easily accessible by heat [37]. Additionally, the third instars have a more mature integument and are very mobile. Conversely, Verghese et al. [30] reported that the first-instar larvae of *B. dorsalis* are the most thermotolerant compared to other immature stages when subjected to a HWT of 48 °C for 60 min. The divergence in results could be attributed to several factors, including differences in the host fruit cultivar and/or the experimental procedures applied [58].

Our HWT schedule of 46.1 °C for 72.63 min (95% Cl: 70.32–74.95) lies within the internationally recommended range of mango disinfestation treatments against fruit flies. For example, the EU recommends a HWT of 65–90 min at 46 °C for *B. dorsalis* for mango, depending on the shape and size of the fruits [23]. The USDA, on the other hand, approves treatment durations ranging from 65 to 110 min at 46.1 °C depending on the weight and shape of the mango fruit [39]. Similarly, many Asian countries recommend that at least 30,000 test individuals be used when testing the efficacy of a treatment and that a mortality of 99.99% (probit 8.72) be established with a 95% Cl [59]. In such cases, the treatment regime will require an exposure time of 69.55 min (95% Cl: 67.38–71.72) at 46.1 °C to control 99.99% of the most heat-tolerant stage (third-instar larvae). 

Heat transfer and its tolerance by fruits have been demonstrated in previous studies to be dependent on several factors, including fruit origin, cultivar, maturity, size, shape, and weight [38,60,61]. These factors may therefore influence the efficacy of hydrothermal treatments and fruit quality post-treatment [21,31]. In the current study, there were no visible signs of heat injuries or accelerated skin color development over the 11-day storage period post-HWT. This finding is comparable to previous trials conducted using various mango varieties. For example, Sharp and Spalding [62] found decreased occurrence of stem-end rot and anthracnose in cv. Tommy Atkins mango subjected to hot water-treatment at 46.1 °C for 65 min. The HWT of the same cultivar at 46.1–46.7 °C for up to 90 min had no visible injury caused by HWT on fruit quality [63]. Similarly, Le et al. [64] demonstrated that cv. Tuu Shien mangoes retained their skin post-HWT of 50 °C for 10 min. Conversely, there were signs of darkened lenticels when cv. Tommy Atkins mangoes were subjected to a HWT of 46 °C for 120 min or 49 °C for 60 min [65]. This indicates that treatment conditions within the optimum range can maintain the color and appearance of the fruit peel; however, those beyond the optimum range are likely to cause abiotic stress leading to heat injuries in mango. 

In the current study, HWT at 46.1 °C for 72.63 did not affect the firmness of the fruit. In a similar study, Hernández et al. [66] showed that hot water phytosanitary treatment against *B. dorsalis* in cv. Tommy Atkin mangoes produced no loss of firmness. However, our results contradict those of Ding and Mijin [67], who reported increased firmness retention in cv. Chok Anan mango fruit subjected to a HWT of 55 °C for 25 min before long-term cold storage. This could be due to several factors, including differences in the physiological maturity of the fruits used in the experiment. The application of HWT at 46.1 °C for 72.63 did not affect the change in the TSS (ºBrix) of the fruits when assessed from the first day and every second day for 11 days after HWT. Kim et al. [68] also found that HWT at 46.1 °C for 70, 90, and 110 min did not affect the change in the soluble solids content of mango 4 days post-treatment. Verghese et al. [30] reported that HWT of mango fruits at 46 °C for 60 min and 48 °C for 60, 75, and 90 min did affect TSS. Similarly, there were no effects on total soluble solids when cv. Tuu Shien mango was subjected to vapor heat treatment at 46.5 °C for 40 min [64]. Conversely, cv. Ataulfo mango increased TSS content during storage at 20 °C for 8 days post-HWT at 47 °C for 5 min [69]. The titratable acidity (TA) of the mango fruits was not affected by HWT 11 days post-treatment. Similar findings have been reported in several studies. Oladele and Fatukasi [70] demonstrated that hot water at 55 °C for 1 and 3 min had no significant effect on TA between treated and control samples 20 days post-treatment. Mansour et al. [71] also reported a lack of effect in TA change in three different mango cultivars (including Tommy Atkins) post-HWT. This study also showed that moisture content was not significantly affected by the treatment in eleven days of storage. This contrasts with Wang et al. [72], who reported a repressed respiration rate in cv. Ivory mango after HWT at 60 °C for 1 min inhibited the respiration rate of cv. Ivory mango and, therefore, reduced moisture loss.

## 5. Conclusions

The volume of mango produced in SSA is higher compared to the volume that can meet export standards, chiefly due to infestation by fruit flies [4,23]. This means that the continent does not fully tap into the lucrative markets abroad and, hence, receives minimum returns from the venture. If post-harvest treatment protocols are developed, standardized, and adopted, the relevant countries will be able to overcome the fruit fly menace. The current study established that a 99.9968% quarantine security level (probit 9) is attained for cv. Tommy Atkins mango at a water temperature of 46.1 °C in 72.63 min, which conforms to the requirements of most mango-importing countries. Additionally, our HWT schedule does not affect cv. Tommy Atkins mango quality and should be commercially adopted for disinfesting Kenyan cv. Tommy Atkins mango infested with *B. dorsalis*. The efficacy of HWT and its effect on mango fruit quality attributes are dependent on several factors, including cultivar, conditions of treatment (temperature and exposure time), environmental factors, fruit maturity, fruit size, and other pre-harvest conditions. Thus, it is critical to design an appropriate post-harvest phytosanitary treatment schedule for each mango cultivar. 

## Figures and Tables

**Table 1:** Development of the immature stages of *B*. *dorsalis* in Tommy Atkins mango fruit following infestation in the laboratory (25 ± 1 °C, 60–70% RH).

	% of Different Developmental Stage			
Day	Eggs	First Instar	Second Instar	Third Instar
1	100	0	0	0
2	9.5	90.5	0	0
3	0	98	2	0
4	0	5.5	94.5	0
5	0	2.5	96.5	1
6	0	0	98	2
7	0	0	39.5	60.5
8	0	0	0.5	99.5
9	0	0	0	100

**Table 2:** Time–mortality relationship for the immature stages of *B. dorsalis* in Tommy Atkins mango fruit after immersion in hot water of 46.1 °C.

Stage	Time (min)	No. of Insects	No. Dead	% Mortality
Egg	8	7014	109	1.55
	15	7032	1496	21.27
	23	6633	3911	58.96
	30	6207	5812	93.64
	38	6372	6366	99.91
	53	7717	7717	100.00
	60	7418	7418	100.00
	68	6760	6760	100.00
1st instar	8	6782	186	2.74
	15	6691	1070	15.99
	23	7134	3011	42.21
	30	6524	4187	64.18
	38	6548	6121	93.48
	53	6489	6489	100.00
	60	7402	7402	100.00
	68	6843	6843	100.00
2nd instar	8	7118	176	2.47
	15	7216	633	8.77
	23	7190	2520	35.05
	30	7300	4869	66.70
	38	7132	6092	85.42
	53	7035	7034	99.99
	60	7487	7487	100.00
	68	7926	7926	100.00
3rd instar	8	5907	132	2.23
	15	5886	568	9.65
	23	5915	2321	39.24
	30	5813	3531	60.74
	38	5734	4534	79.07
	53	5893	5781	98.10
	60	5924	5892	99.46
	68	6034	6034	100.00

**Table 3:** Probit model estimates of time required to achieve 50%, 90%, 99%, 99.9%, 99.99%, and 99.9968% mortality of different immature stages of *Bactrocera dorsalis* in Tommy Atkins mango after HWT of 46.1 °C.

Stage	No. of Insects	LT_50_	LT_90_	LT_99_	LT_99.9_	LT_99.99_	LT_99.9968_
Egg	55,153	20.90 (20.52–21.28)	28.91 (28.34–29.49)	35.45 (34.56–36.33)	40.22 (39.10–41.35)	44.16 (42.82–45.49)	45.90 (44.47–47.33)a
1st instar	54,413	24.81 (24.34–25.27)	36.20 (35.43–36.98)	45.50 (44.29–46.70)	52.29 (50.74–53.84)	57.88 (56.05–59.71)	60.36 (58.40–62.32)b
2nd instar	58,404	27.28 (26.81–27.75)	38.71 (37.90–39.52)	48.03 (46.78–49.27)	54.84 (53.25–56.43)	60.45 (58.57–62.39	62.93 (60.93–64.94)b
3rd instar	47,106	28.35 (27.81–28.89)	42.55 (41.61–43.48)	54.12 (52.68–55.56)	62.58 (60.75–64.42)	69.55 (67.38–71.72)	72.63 (70.32–74.95)c

The values in the brackets () indicate critical limits, CL. Lethal times in the last column (LT_99.9968_) followed by a different lowercase letter are significantly different (α = 0.05).

**Table 4:** Mortality of third-instar larvae of *Bactrocera dorsalis* in Tommy Atkins mango fruit subjected to hot water of 46.1 °C for 72.63 min.

		Hot Water Treatment				Untreated Control			
Replicate	No. of Fruits	No. of Insects	No. Dead	No. Alive	% Mortality	No. of Insects	No. Dead	No. Alive	% Mortality
1	160	9159	9159	0	100	9448	228	9220	2.41
2	160	7142	7142	0	100	7266	151	7115	2.08
3	160	7339	7339	0	100	7451	148	7303	1.99
4	160	7483	7483	0	100	7522	122	7400	1.62
5	160	6957	6957	0	100	7192	216	6976	3.00
6	160	7825	7825	0	100	7838	318	7520	4.06
7	160	6583	6583	0	100	6625	284	6341	4.29
8	160	6632	6632	0	100	6680	263	6417	3.94
Total	1280	59,120	59,120	0	100	60,022	1730	58,292	2.92

## Data Availability

All data sets presented in this study are included in the article and can be availed by the authors upon reasonable request.
